# A Randomized Clinical Trial to Evaluate the Efficacy and Safety of the ACTLIFE Exercise Program for Women with Post-menopausal Osteoporosis: Study Protocol

**DOI:** 10.3390/ijerph17030809

**Published:** 2020-01-28

**Authors:** Laura Bragonzoni, Giuseppe Barone, Francesco Benvenuti, Veronica Canal, Claudio Ripamonti, Sofia Marini, Laura Dallolio

**Affiliations:** 1 Department of Life Quality Studies, University of Bologna; Campus Rimini, Corso d’Augusto, 237, 47921 Rimini, Italy; Laura.bragonzoni4@unibo.it (L.B.); benvefrancis@gmail.com (F.B.); sofia.marini2@unibo.it (S.M.); 2 Unit of Hygiene, Public Health and Medical Statistics, Department of Biomedical and Neuromotor Science, University of Bologna, Via San Giacomo, 12, 40138 Bologna, Italy; canal.veronica@gmail.com (V.C.); laura.dallolio@unibo.it (L.D.); 3 Center for Osteoporosis and Bone Metabolic Disease, Rizzoli Orthopaedic Institute, Via G.C. Pupilli,1, 40136 Bologna, Italy; claudio.ripamonti@ior.it

**Keywords:** post-menopausal osteoporosis, physical activity, quality of life, exercise, gym training, home training

## Abstract

Osteoporosis (OP) is a systemic disease of the skeleton characterized by increased risk of fracture. There is a general consensus on the efficacy of physical activity in the prevention of bone loss, falls and fractures, but there is no agreement on the best setting to exercise. The aim of the study is to evaluate the efficacy of a 12-months exercise protocol for women with post-menopausal OP when administered as individual home training (IHT) versus gym group training (GGT). The study is a randomized trial with two parallel groups. Sedentary patients with primary post-menopausal osteoporosis are recruited at the Istituto Ortopedico Rizzoli of Bologna. In the first group, the 12-month ACTLIFE program is performed as IHT, while in the second as GGT. The program is aimed at improving joint mobility, muscle force, balance, motor coordination and endurance. The study is single blinded. Patients are assessed at baseline and after 6 and 12 months. The primary outcome is the modification of quality of life measured with the Short Osteoporosis Quality of Life Questionnaire (ECOS-16). The findings of this study will highlight advantages and disadvantages of exercising in the two different settings and provide evidence on how to increase physical activity in osteoporotic women.

## 1. Introduction

Osteoporosis (OP) is a systemic disease of the skeleton characterized by reduced mass and deterioration of micro-architecture of the bone that is accompanied by an increased risk of fracture with consequent pain, decreased physical and social functional capacity and quality of life (QoL) [1,2,3,4]. There is a general consensus on the efficacy of physical activity in the prevention of bone loss, falls and, consequently, fractures [5,6]. A number of randomized controlled trials proved the efficacy of exercise programs versus no exercise, sham programs or drugs in women with OP [7]. Exercise programs were administered individually as home training (IHT) or in gyms as group training (GGT). However, to our knowledge, there are no studies comparing the effects of an exercise program specifically designed for women with post-menopausal OP when administered as IHT versus GGT. There are studies that compared the benefits of the two settings for exercise programs designed for the prevention of other conditions. Exercise aimed at improving pain and function in chronic nonspecific low back pain is more effective when delivered in gyms with supervision [8,9]. Studies compared the Otago exercise programs for fall prevention proved a better efficacy in group activity for variables related to physical and mental health [10,11].

Regular participation in a physical activity program is vitally important for the geriatric population to prevent decline in mobility function [12]. However, adherence to an exercise program is problematic in all age groups, but particularly among older adults. For instance, a meta-analysis of 127 exercise interventions for older adults demonstrated that, within the first three to six months, 40–65% of the participants will drop out [13]. In a previous study, we investigated predictive factors of improved back-pain status among older adults with chronic back pain participating in a 12-month GGT program [14]. We found that adherence was the key predictor of improved back pain. Adherence, in turn, was independently associated to accessibility to gyms. Thus, the question arose whether IHT could be a valid alternative of GGT since, from the theoretical point of view, it could overcome problems related to accessibility to gyms or timetable rigidity.

This paper aimed at presenting the protocol of a randomized trial for evaluating the efficacy of a physical activity program (12-month duration) designed to improve the quality of life in women with post-menopausal OP when administered IHT or GGT. We relied on the most recent scientific evidence in this field, to develop the exercise program designed to improve the quality of life in this population [15,16,17]. We hypothesize that efficacy and safety of the exercise program are equal when administered as GGT or IHT. However, differences in terms of intensity, supervision, progression and adherence between the two groups may have different impact on the outcome measures.

## 2. Materials and Methods

This study is carried out within the project “Physical ACTivity: the tool to improve the quality of LIFE in osteoporosis people” (ACTLIFE) funded by European Commission within the Erasmus+ Sport program (Grant Agreement N2017-2128/001-001). The study was approved from the Local Ethics Committee (Comitato Etico Indipendente di Area Vasta Emilia Centro, CE-AVEC) of the Emilia-Romagna Region (reference number AVEC: EM601-2019_696/2018/Sper/IOR_EM2). The trial was registered in ClinicalTrial.Gov (NCT04179903).

### 2.1. Study Design

The study is a randomized trial with two parallel groups: in the first group the 12-month ACTLIFE exercise program is performed as IHT, while in the second as GGT. It is single blinded since professionals who evaluate patients are not aware to which exercise group patients are assigned. Patients are assessed at baseline and after 6 and 12 months.

### 2.2. Participant Recruitment

Sedentary patients with primary post-menopausal OP are recruited by the Centro Osteoporosi e Malattie Metaboliche dello Scheletro (COMMS) of the Istituto Ortopedico Rizzoli of Bologna, Italy. The participation to the study was proposed to all women attending COMMS for a medical visit who met the inclusion criteria. In addition, to facilitate patients’ recruitment, the study was advertised on local media requesting the women who might be interested to contact the COMMS for a preliminary evaluation.

A letter was sent to a participant’s home in an anonymous envelope, asking the patient to contact the person in charge of the study (or his staff). The letter described the scientific purposes, not specifying the type of study.

During the study, all pharmaceutical treatments, and their modifications were recorded. Patients were recommended to adhere pharmacological treatment for OP, as prescribed.

### 2.3. Inclusion and Exclusion Procedures

The inclusion/exclusion criteria (Table 1) were identified during a preliminary medical visit and functional assessment. If eligible, patients were requested to sign the informed consent form. Subsequently, they were recruited and given an individual study code (number based on the order of inclusion in the study). Personal data were recorded only on the informed consent form together with the individual study code. Patients were identified only with the individual study code in all the other forms and databases used in the study.

### 2.4. Description of Procedure and Randomization

The professional who enrolled the patients assigned them to the GGT or IHT group after contacting a dedicated person responsible of the maintenance of the randomization list. The randomization list was defined using the random numbers generator available on the web site of the Emilia-Romagna Region (http://wwwservizi.regione.emilia-romagna.it/generatore/). For each of the two groups (IHT and GGT), 26 numbers from 1 to 52 were generated.

### 2.5. Allocation, Concealment and Blinding

The random assignment to IHT or GGT groups was performed by different personnel than those who performed the assessments at baseline, six and 12 months.

The list of allocation of the patients to one of the two groups was kept locked and separated from the rest of the material used to collect patients’ information. At any time, professionals who performed the assessments were not aware to which group patients had been assigned. Patients were clearly instructed not to reveal to the trainer who performed the assessment which exercise group they were in.

### 2.6. Sample Size

Sample size was estimated considering the questionnaire ECOS-16 [21] as a primary outcome measure of the study. From published evidence, ECOS-16 has a standard deviation of 0.8 at final follow-up assessment and a minimal clinically important difference of 0.69. This leads to an estimated effect size of 0.863. Considering an alpha error of 0.05 and a power of at least 0.8, the minimum size of the sample was estimated in 18 patients per group, for a total of 36 patients. Considering a 15% drop-out (estimated on the basis of the experience of a previous study focused on patients with OP vertebral fractures [16]), preferring to be even more conservative, we estimated an appropriate sample size of 26 patients for each group, for a total number of 52 participants. Power analysis was carried out with G*Power 3.1.9.2.

### 2.7. Data Collection and Measures

Instruments to record primary and secondary outcome measures and the time of their used are summarized in Table 2.

#### 2.7.1. Primary Outcome

The primary outcome was quality of life (QoL) measured with the Short Osteoporosis Quality of Life Questionnaire (ECOS-16) [21], specifically designed to measure the health related QoL in post-menopausal women with OP. It was based on the combination of Osteoporosis Quality of Life Questionnaire and Quality of life questionnaire of the European Foundation for Osteoporosis. Specific-disease instruments have been proved to be of paramount importance to evaluate responses to treatments [22,23]. Validity and reliability of ECOS-16 had also been previously proved for the Italian version of the questionnaire [24]. This measure was repeated at baseline, 6 and 12 months.

#### 2.7.2. Secondary Outcomes

Secondary outcomes were measured, which were recognized to influence QoL.

Body Mass Index (BMI) was calculated as the ratio of body weight squared (kg/m2) and the impedance measurements were performed with bioimpedance analysis (BIA 101 Anniversary®, Akern, Florence, Italy) [25,26,27] to evaluate body composition (fat mass, muscle mass and bone mass).

The WHO Disability Assessment Schedule (WHODAS) [28] is composed of 36 items to represent the six activity and participation domains (cognition, mobility, self-care, getting along, life activities, participation). The Italian version of the instrument has been previously validated [29,30].

Falls Efficacy Scale International (FES-I) [31] is a 16 items questionnaire to assess fear of falling during simple and complex motor and social activities; this instrument has also been validated in Italian [32]. Furthermore, history of fall was recorded by self-reported falls (number) in the previous three months.

Physical Activity Scale for Elderly (PASE) [33,34], which has also been validated in Italian [35], is a scored survey designed specifically to measure the weekly physical activity in adult and aged population. Its score combines information on leisure, household and occupational activity.

Individuals’ functional capacity is estimated by measuring several domains. Gait performance was evaluated by the 6-Minute Walk Test, which has been proven to be a valid and reliable instrument [36,37]. It is a test very easy to administer and allows to measure patients’ residual functional capacity in a number of pathological conditions [38,39,40,41], including OP [42].

To better understand individuals’ functional capacity modifications, we also included measures of standing balance, muscle force and joint mobility, which are considered prerequisites of motor functioning. Standing balance is estimated by the validated Stability Index [43] for both right and left lower limbs. For these measures, the Delos Postural Proprioceptive System® (Delos S.r.l., Torino) were used. For muscle force, the hand grip [44,45] was bilaterally measured by Hydraulic Hand Jamar Dynamometer®. This measure has been strongly associated to frailty in elderly population [44]. The occiput-wall distance was used to estimate postural alignment [46]. The measure was the distance between the head and a wall, while the subject stood with their heels touching a wall. Sit-and-reach tests were used as indirect measures to assess hamstring and low back flexibility. This test measured the fingertips-to-tangent feet distance of the subjects when they were sitting on a chair [47].

Finally, joint mobility of shoulder, hip and knee, which are fundamental prerequisites of healthy and safe motor behavior, were assessed by routinely used clinical measures [48,49]. Shoulders were evaluated in sitting position, requesting the subject to actively flex both the extended arms while holding a 1.2 m stick. The minimum distance between the hands was measured when the stick was over the vertex of the head. When subjects were unable to bring the stick in the requested position, even with the largest holding, the stick length was conventionally attributed. Hip and knee ROMs were bilaterally evaluated in lying position asking an active flexion of one joint while the other was kept in neutral position. Maximum flexion degree is reported.

Patients’ adherence to the exercise program was recorded by home or gyms weekly logs and measured as the percentage of exercise sessions actually performed/total number of scheduled exercise sessions.

The reasons of interruption and abandons were carefully evaluated during the study period. All patients were also instructed to contact the project staff to communicate adverse clinical events or other reasons of non-participation, whenever this was deemed necessary. Finally, patients’ satisfaction is assessed at the end of the study by a specifically designed questionnaire based on seven-point Likert scale [50,51] investigating the perceived quality and self-efficacy of the ACTLIFE program.

### 2.8. Intervention

The exercise program was administered to both GGT and IHT groups. It was aimed at improving joint mobility, muscle force, static and dynamic balance, motor coordination and endurance. For each subject of both groups, the program was structured in 2-days/week 1-hour sessions and lasts 12 months.

Moreover, subjects were requested to choose an additional third day of the week to carry out at least one of the following activities: brisk walking, cycling or swimming to reach the weekly amount of exercise of 150 minutes recommended by World Health Organization [52].

Each session was structured in the following sections: warm-up, strength, balance, flexibility and cool down. The exercise program was redundant to allow the trainer to adapt the exercise program to the participants’ needs and preferences. The protocol defines the strategies to instruct patients and to check the correct and safe execution of the motor tasks and the criteria for varying workload and number of repetitions to adapt to each individual functional capacity. For both groups, the exercise program was administered by a graduate trainer in Science and Techniques of Preventive and Adapted Physical Activity.

GGT was performed in adequately equipped gyms, which had stipulated formal agreements with the University of Bologna under the direct supervision of a graduate trainer. Every 6-8 weeks, the trainer upgraded the exercise program on the basis of the improvement obtained by the gym group.

For the IHT group, the trainer explained to the participant the physical activity (PA) program to be performed at home in one or more individual sessions. At the end of these initial instructional session(s), the participant was also given educational material on the purpose of the exercises and how to perform them correctly. Participants were requested to strictly adhere to the given instructions. Subsequently, the trainer contacted the IHT participants at pre-established time intervals: once a week for the initial two weeks and twice a month for the following 11 months to encourage participants to exercise regularly and to get information on the health status. Every 6-8 weeks, an appointment was scheduled to review the exercise program. All contacts of trainers with IHT participants were recorded.

Monthly logbooks were used to record the adherence to the exercise program by noting down the execution of this session both at home and in the gym. For the former, a recording was made by the participant, for the latter by the trainer. The logbooks were subsequently collected by research staff or returned by mail with pre-paid envelopes. Finally, a telephone number and a week time schedule in which a trainer was available for further explanations and suggestions were given to them.

### 2.9. Statistical Analysis

The qualitative variables are summarized in terms of frequency, the quantitative ones in terms of mean and standard deviation for both groups and for the three times of assessment. For the analysis of the results, the principle of intention to treat is used, adjusting for adherence to the exercise program.

To compare the general characteristics between the two groups, the Student’s t-test is used for parametric quantitative variables, the Mann Whitney test for non-parametric variables and Chi-square test for qualitative dichotomous ones.

To compare the changes between the two settings among baseline and follow-up assessments the analyses of variance for repeated measures followed by of Sidak post-hoc comparisons tests for paired samples were used for the quantitative variables and the Friedman test, followed by the Wilcoxon test for paired samples with Bonferroni correction for non-parametric ones.

## 3. Discussion

Several lines of evidence have consistently proven the importance of regular participation in specific exercise programs to prevent/minimize the osteoporotic bone deterioration and its consequences in post-menopausal women [5,6,7]. However, to the best of our knowledge, no study has yet examined exercise programs for women with post-menopausal OP when administered as IHT or GGT. From a theoretical point of view, each setting may have advantages and disadvantages [10,12,53]. Older frail people may have a reduced functional capacity and have difficulty following the rhythms of group activity. Travel to and from the gym may be problematic, especially during the winter months, and/or require regular, often unavailable, commitments from family members or caregivers. For women with good health and functional status attending gyms at scheduled intervals may be problematic due to family or work obligations. On the other hand, the activity in the gym has a greater level of supervision and, probably, will ensure a greater amount of exercise to actually be performed. Numerous factors have been demonstrated as barriers to regular exercise in older adults, including perceived poor health, poor self-confidence, low motivation and perceived exercise enjoyment [54]. Experts in group dynamics have suggested that participation in regular group activities can lead to true behavior change through a pathway of social interaction, group bonding and behavior imitation [55]. In other patient populations (i.e., patients with cancer), group exercise has been shown to result in improved quality of life, greater self-confidence, increased motivation and a sense of camaraderie with other participants [56]. Finally, clinicians may consider the exercise safer when performed under supervision of a professional trainer and, therefore, more willing to advice GGT than IHT.

Some limitations of the present protocol must be considered because they may influence the interpretations of the findings of the study. The study did not consider a control group performing sham or no activity since we focused on comparing GGT versus IHT. Therefore, we cannot empirically prove the efficacy of the exercise program administered either as GGT or IHT. However, the vast and substantial published evidence [1,2,3,4,5,6,7] have led the medical community to recommend patients with OP physical activity extensively. Thus, the implementation of a control group appeared to the authors to be not feasible and not acceptable from an ethical point of view. Participants are post-menopausal women recruited on the basis of inclusion/exclusion criteria (Table 1). Most of them may be under pharmacological treatment not only for osteoporosis but also for other chronic conditions with high prevalence in post-menopausal women. We cannot exclude that both associated chronic conditions and pharmacological treatments may influence the outcome of the study. Therefore, data related to comorbid conditions and pharmaceutical treatment were recorded at baseline and during the entire study.

## 4. Conclusions

Although guidelines for OP prevention recommend to exercise regularly, there are no specific indications on the best setting to exercise. The results of this study could be relevant for future indication of the best setting and strategy to ensure the adherence to the physical activity and add to the current evidence base for clinicians, exercise trainers and policy makers.

## Figures and Tables

**Table 1:** Inclusion and exclusion criteria.

Inclusion Criteria	Exclusion Criteria
▪ Signed informed consent ▪ Post-menopausal women aged ≥ 40 years ▪ Lumbar spine or femur T-score ≤ -2.5 ▪ SPPB* ≥ 6 ▪ Having exercised less than 30 minutes per week in the last 6 months	▪ Secondary osteoporosis ▪ Severe impairment of communicative and/or sensorial functions ▪ Heart failure (NYHA** class ≥ 2) ▪ Unstable angina ▪ Pulmonary disease requiring oxygen therapy ▪ Symptomatic orthostatic hypotension ▪ Hypertension in poor pharmacologic control (diastolic >95 mmHg, systolic >160 mmHg) ▪ Previous implant of prosthesis at upper or lower limbs ▪ Relevant neurological condition impairing motor or cognitive function ▪ Any other condition that the General Practitioner considers to contraindicate the participation in an exercise program of moderate intensity

* SPPB = Short Physical Performance Battery [18,19]; **NYHA = New York Heart Association [20].

**Table 2:** Outcome assessment.

Outcome Assessment	Baseline (T0)	6 Months (T1)	12 Months (T2)
ECOS-16	x	x	x
BMI	x		x
BIA	x		x
WHODAS	x		x
FES-I	x	x	x
Falls	x	x	x
PASE	x	x	x
6MWT	x	x	x
Delos	x		x
Handgrip	x	x	x
Occiput-wall distance	x	x	x
Chair sit and reach	x	x	x
Range of motion (shoulder, hip, knee)	x	x	x
Adherence		x	x

## Abbreviations

6MWT	6-Minute Walk Test
ACTLIFE	Physical Activity: the tool to improve the quality of LIFE in osteoporosis people
BMD	Bone Mineral Density
BMI	Body Mass Index
ECOS-16	Evaluación De La Calidad De Vida En La Osteoporosis (Assessment of Health Related Quality Of Life In Osteoporosis)
FaME	Falls Management Exercise
FES-I	Falls Efficacy Scale International
GGT	Gym Group Training
IHT	Individual Home Training
OEP	Otago Exercise Program
PA	Physical activity
PASE	Phisical Activity Scale for Elderly
QoL	Quality of Life
OP	Osteoporosis
WHODAS	WHO Disability Assessment Schedule
